# The seasonality of cholera in sub-Saharan Africa: a statistical modelling study

**DOI:** 10.1016/S2214-109X(22)00007-9

**Published:** 2022-04-21

**Authors:** Javier Perez-Saez, Justin Lessler, Elizabeth C Lee, Francisco J Luquero, Espoir Bwenge Malembaka, Flavio Finger, José Paulo Langa, Sebastian Yennan, Benjamin Zaitchik, Andrew S Azman

**Affiliations:** a Department of Epidemiology, Johns Hopkins Bloomberg School of Public Health, Johns Hopkins University, Baltimore, MD, USA; b Department of Earth and Planetary Sciences, Johns Hopkins University, Baltimore, MD, USA; c Unité d'Épidémiologie Populationnelle, Geneva University Hospitals, Geneva, Switzerland; d Department of Epidemiology, Gillings School of Global Public Health, and University of North Carolina Population Center, University of North Carolina at Chapel Hill, Chapel Hill, NC, USA; e Gavi, the Vaccine Alliance, Geneva, Switzerland; f Center for Tropical Diseases and Global Health, Université Catholique de Bukavu, Bukavu, Democratic Republic of the Congo; g Epicentre, Paris, France; h Instituto Nacional de Saúde, Maputo, Mozambique; i Surveillance and Epidemiology, Nigeria Centre for Disease Control, Abuja, Nigeria; j Institute of Global Health, Faculty of Medicine, University of Geneva, Geneva, Switzerland

## Abstract

**Background:**

Cholera remains a major threat in sub-Saharan Africa (SSA), where some of the highest case-fatality rates are reported. Knowing in what months and where cholera tends to occur across the continent could aid in improving efforts to eliminate cholera as a public health concern. However, largely due to the absence of unified large-scale datasets, no continent-wide estimates exist. In this study, we aimed to estimate cholera seasonality across SSA and explore the correlation between hydroclimatic variables and cholera seasonality.

**Methods:**

Using the global cholera database of the Global Task Force on Cholera Control, we developed statistical models to synthesise data across spatial and temporal scales to infer the seasonality of excess (defined as incidence higher than the 2010–16 mean incidence rate) suspected cholera occurrence in SSA. We developed a Bayesian statistical model to infer the monthly risk of excess cholera at the first and second administrative levels. Seasonality patterns were then grouped into spatial clusters. Finally, we studied the association between seasonality estimates and hydroclimatic variables (mean monthly fraction of area flooded, mean monthly air temperature, and cumulative monthly precipitation).

**Findings:**

24 (71%) of the 34 countries studied had seasonal patterns of excess cholera risk, corresponding to approximately 86% of the SSA population. 12 (50%) of these 24 countries also had subnational differences in seasonality patterns, with strong differences in seasonality strength between regions. Seasonality patterns clustered into two macroregions (west Africa and the Sahel *vs* eastern and southern Africa), which were composed of subregional clusters with varying degrees of seasonality. Exploratory association analysis found most consistent and positive correlations between cholera seasonality and precipitation and, to a lesser extent, between cholera seasonality and temperature and flooding.

**Interpretation:**

Widespread cholera seasonality in SSA offers opportunities for intervention planning. Further studies are needed to study the association between cholera and climate.

**Funding:**

US National Aeronautics and Space Administration Applied Sciences Program and the Bill & Melinda Gates Foundation.

## Introduction

Despite being one of the oldest known infectious diseases, cholera—typically caused by toxigenic *Vibrio cholerae* bacteria of serogroup O1—still causes between 1 and 4 million cases per year.^1^ Most cholera cases reported to WHO between 1996 and 2018, excluding the 2010 Haitian and 2017 Yemen epidemics, have occurred in sub-Saharan Africa (SSA), which also has the highest case-fatality rates (eg, 2% in 2018).^2^ An estimated 87 million people in SSA live in districts with high cholera incidence.^3^ Cholera transmission spans the endemic–epidemic spectrum across SSA, with large heterogeneity in transmission characteristics across time and space.^4^, ^5^ Tailoring cholera prevention and control programmes to local epidemiological characteristics might be one efficient way to reach global targets for cholera control,^6^ although detailed systematic descriptions across broad geographies remain sparse.

Seasonality is one important aspect of cholera epidemiology, and cholera exhibits strong seasonal patterns in countries on the Bay of Bengal. The seasonal patterns of cholera in coastal and estuarine areas in this region have been linked in part to the ecology of *V cholerae* in its natural brackish water habitats.^7^, ^8^ Case studies from individual countries over short time periods in SSA have shown diverse seasonal patterns in cholera occurrence,^9^, ^10^, ^11^ although these fragmented descriptions have limited use in furthering our understanding of cholera dynamics and for global or regional public health planning. One of the major challenges hindering detailed large-scale descriptions of cholera seasonality has been the absence of unified, fine-scale spatial and temporal resolution datasets on cholera occurrence.

Understanding seasonal variations in transmission of infectious diseases, such as cholera, has direct implications for improving surveillance systems and tailoring control and elimination efforts.^12^, ^13^ A better understanding of seasonality could allow for adaptive cholera surveillance and testing protocols, improvements in cholera risk assessments, and improvements in planning cholera-prevention activities, and it could be used to help trigger local disease control activities. A detailed understanding of cholera seasonality could also enhance our ability to disentangle the links between cholera, climate, and human behaviour.

**Research in context**

**Evidence before this study**

We searched PubMed for previous studies on Nov 15, 2021, with no language or date restrictions using the search terms [“cholera*” AND “season*” AND (“Africa*” OR “Angola” OR “Burundi” OR “Benin” OR “Burkina Faso” OR “Botswana” OR “Central African Republic” OR “Côte d’Ivoire” OR “Cameroon” OR “Democratic Republic of the Congo” OR “Republic of Congo” OR “Djibouti” OR “Eritrea” OR “Ethiopia” OR “Gabon” OR “Ghana” OR “Guinea” OR “Gambia” OR “Guinea-Bissau” OR “Equatorial Guinea” OR “Kenya” OR “Liberia” OR “Madagascar” OR “Mali” OR “Mozambique” OR “Mauritania” OR “Malawi” OR “Namibia” OR “Niger” OR “Nigeria” OR “Rwanda” OR “Sudan” OR “Senegal” OR “Sierra Leone” OR “Somalia” OR “South Sudan” OR “São Tomé and Príncipe” OR “Swaziland” OR “Chad” OR “Togo” OR “Tanzania” OR “Uganda” OR “South Africa” OR “Zambia” OR “Zimbabwe”)]. Two additional known articles not identified by our PubMed search were added, one on the seasonality of cholera in Kenya and one on the epidemiology of cholera in west Africa. Studies were included if they focused on the epidemiology of *Vibrio cholerae* O1 or O139, covering one or multiple countries in sub-Saharan Africa (SSA) for at least 2 years with reported cases. We identified 140 studies, of which 36 met our inclusion criteria. Of these, four were reviews, three were regional or global studies, and 29 focused on specific countries. Local-level seasonality studies were identified in Burundi (one), Côte d’Ivoire (one), Cameroon (two), Democratic Republic of the Congo (three), Ghana (one), Guinea-Bissau (one), Kenya (one), Mozambique (three), Nigeria (one), Senegal (one), Somalia (one), South Sudan (one), Togo (one), Tanzania (four), Uganda (two), South Africa (one), and Zambia (four). These local studies mainly consisted of epidemiological descriptions of cholera incidence at either the national or first administrative unit scale, covering between 2 years and 31 years of data. Most of these studies found seasonality in cholera incidence, with different patterns between countries. Two regional studies in west Africa found seasonal patterns in cholera incidence in the second part of the calendar year, with evidence for synchrony with rainfall patterns. Finally, one global study assessing the period of 1975–2005 found evidence for a latitudinal gradient of seasonality strength with weaker seasonality around the equator.

**Added value of this study**

Although most local-level and regional studies have found evidence for cholera seasonal patterns in SSA, gaps remain for some countries, and a continental-scale investigation is currently needed. By using a large database of cholera incidence, we evaluated and characterised cholera seasonality at a subnational scale in 34 countries of SSA for which sufficient data was available. We showed that cholera is seasonal in the majority of countries (24 [71%] of 34), with subnational heterogeneity in half of them (12 [50%] of 24). Our results enable the description of cholera seasonality at the continental level and the exploration of associations between cholera incidence and hydroclimatic variables.

**Implications of all the available evidence**

This work establishes the extent and strength of cholera seasonality in SSA, providing a basis on which to ground strategic decisions on large-scale intervention allocation, as well as future work on the climatic and non-climatic drivers of cholera seasonality in SSA.

In this study, we aimed to develop an almanac of cholera seasonality across SSA, allowing for a subnational understanding of how cholera risk varies throughout the year. We then used these results to explore the correlation between hydroclimatic variables and seasonality across the continent.

## Methods

### Cholera data

The cholera incidence data used in these analyses come from a large database curated by Johns Hopkins University on behalf of the Global Taskforce on Cholera Control (GTFCC). Data consist of suspected and confirmed cholera case reports from various ministries of health, WHO, Médecins Sans Frontières, ProMED, ReliefWeb, scientific literature, and publicly available epidemiological reports. Suspected-case definitions across counties and time periods do vary but are largely based on the recommended WHO and GTFCC case definitions.^14^ Data resolution spanned multiple temporal (from day to multi-year) and spatial (from health zone or area to country) scales. Sub-monthly cholera incidence data were aggregated to the monthly time scale. Data were aggregated to both the first-level and second-level administrative units^15^ for separate analyses at these spatial scales. Details on data aggregation and availability per country are given in the appendix (pp 1–2).

For our primary outcome variable, we defined a binary indicator of reported excess cholera occurrence, defined as above-average incidence. The indicator of excess cholera equals one when the monthly cholera incidence rate for a given administrative unit exceeds the 2010–16 estimated mean monthly incidence rate,^3^ or it equals zero otherwise. In sensitivity analyses, we explored two alternative definitions of cholera occurrence: one or more suspected cases in a given month and ten or more suspected cases in a given month. Of the 44 countries in SSA, we included only those for which cholera was reported at a monthly or sub-monthly resolution data for at least 3 years of the study period. On this basis, we exclude ten countries with insufficient data (Botswana, Republic of the Congo, Eritrea, Gabon, The Gambia, Equatorial Guinea, Mauritania, Rwanda, São Tomé and Príncipe, and Eswatini), comprising about 1·2% of the SSA population, from the analysis due to absence of or insufficient sub-yearly data.

### Model of cholera occurrence

We developed a modelling framework to infer the monthly relative risk of cholera occurrence at the administrative unit level, accounting for inter-annual variability and spatial dependence. We built upon a Bayesian model commonly used for areal count data, which consists of a logistic regression with random effects for the month of the year (assumed to be temporally autocorrelated) and year, as well as a combination of spatially correlated and uncorrelated random effects at the administrative unit level with an intrinsic conditional autoregressive prior probability distribution.^16^ Models were fit to each country separately, integrating multiple observations from different data sources covering the same month and administrative units by assuming that the combined reports of cholera excess occurrence follow a binomial distribution (eg, if two data sources reported excess cholera for a given administrative unit and month and a third did not, the data would be treated as two successes in three binomial trials). Observations covering multiple second-level administrative units, multiple months, or both were included in the analysis by computing their corresponding probabilities of excess cholera occurrence (appendix p 4).

We considered a suite of different models that allowed for varying levels of flexibility in the seasonality of cholera within each country. First, we considered a model that has a single seasonal pattern per country, without assuming any shape (eg, seasonality can be flat, unimodal, or multimodal). We then expanded this model to allow for two seasonal patterns within each country using a two-group mixture model with spatially auto-correlated grouping parameters. Within the mixture model, each administrative unit has a specific probability (ie, mixture probability) of having one of two national seasonality patterns. We jointly estimated the two seasonality patterns and the mixture probabilities. We also extended these models to include inference on the start of the cholera year (eg, the starting month that allowed for the best fit of both the seasonal and annual random effects; more details in the appendix, p 4), which does not necessarily align with the calendar year. We fitted a total of five models for each country: no seasonality (null model), a single seasonal pattern (base model), a single seasonal pattern with a distinct cholera year (offset model), two seasonal patterns (mixture model), and two seasonal patterns with a distinct cholera year (mixture offset model). The primary inference outcome in all models, except for the null, were the odds ratios of observing excess cholera in each calendar month. Models were fitted using Markov Chain Monte Carlo methods with Rstan (version 2.7). We ran four chains for each model with 1000 iterations (after 1000 warm-ups) and assessed chain convergence using the Gelman–Rubin statistic (R-hat).^17^ For each country, we selected the best-fit model on the basis of leave-one-out cross-validation.^18^ Technical details are provided in the appendix (pp 3–6).

### Seasonality index

To characterise the strength of cholera seasonality, we computed a seasonality index for each administrative unit. This index was defined as the proportion of cholera risk that occurs during the cholera seasonality peak. The proportion was computed for each administrative unit as the sum of the mean predicted probabilities of occurrence within a 3-month window centred on the month of peak seasonality divided by the total sum of mean predicted probabilities. We subdivided administrative units into four categories of seasonality strength: those where cholera risk in the 3 months surrounding the risk peak accounted for less than 30%, 30–50%, 50–70%, and more than 70% of the total excess cholera risk.

### Spatial seasonality grouping

To characterise distinct seasonal cholera patterns across SSA, we used a soft-K-means clustering algorithm to infer groupings of administrative units with similarly shaped monthly relative risk curves.^19^ We implemented this algorithm in a Bayesian framework that accounts for spatial correlation between areas. We fitted models with two to ten seasonal groups (K) and compared model performance on the basis of the estimated log-predictive density.^18^

### Seasonality and climatology

We explored the association between the monthly relative risks of cholera and a suite of matched monthly hydroclimatic variables. These included 10 km resolution estimates of 2-m-height air temperature generated by topographically downscaling the Modern Era Reanalysis for Research and Applications version 2 (MERRA-2; US National Aeronautics and Space Administration [NASA]),^20^ monthly precipitation totals drawn from the Climate Hazards InfraRed Precipitation with Stations version 2 (CHIRPSv2)^21^ dataset, and 1/12° (about 10 km) resolution estimates of monthly mean and maximum inundated area from the FloodScan multi-satellite product.^22^ Estimates of additional hydrological variables (soil moisture, runoff, and streamflow) were generated with an offline simulation of the Noah-Multi-Parameterization Land Surface Model^23^ coupled to the Hydrological Modeling and Analysis Program,^24^ using MERRA-2 and CHIRPSv2 as meteorological forcing. We quantified the associations between the monthly risk of cholera and hydroclimatic variables with Spearman correlation coefficients considering different lags between cholera and hydroclimatic variables (0–2 months).

Data used in these analyses were not identifiable and this work was deemed to be non-human subjects research by the Johns Hopkins Bloomberg School of Public Health's institutional review board. Code, results, and a minimal dataset are available online.

### Role of the funding source

The funder had no role in the study design, data collection, data analysis, data interpretation, or writing of the report.

## Results

Our analysis dataset included 236 741 records of cholera incidence from 34 countries in SSA between 1970 and 2021, spanning 2496 distinct second-level administrative units and 426 first-level administrative units (97·7% of records since 2000; appendix p 3). Of the 236 741 records, 221 556 (93·6%) were monthly or submonthly observations.

We found statistical evidence in 24 (71%) of 34 countries (86% of the SSA population) to support the hypothesis that excess cholera occurrence follows a distinct seasonal pattern (that we refer to as seasonality; appendix pp 6–9). The countries for which a model with no seasonality was best supported were Burundi, Central African Republic, Djibouti, Ghana, Liberia, Madagascar, Namibia, Senegal, Togo, and Zimbabwe. Among countries with seasonal cholera, 12 (50%) of 24 had evidence for within-country differences in seasonal cholera patterns (appendix pp 6–9).

**Figure 1:**
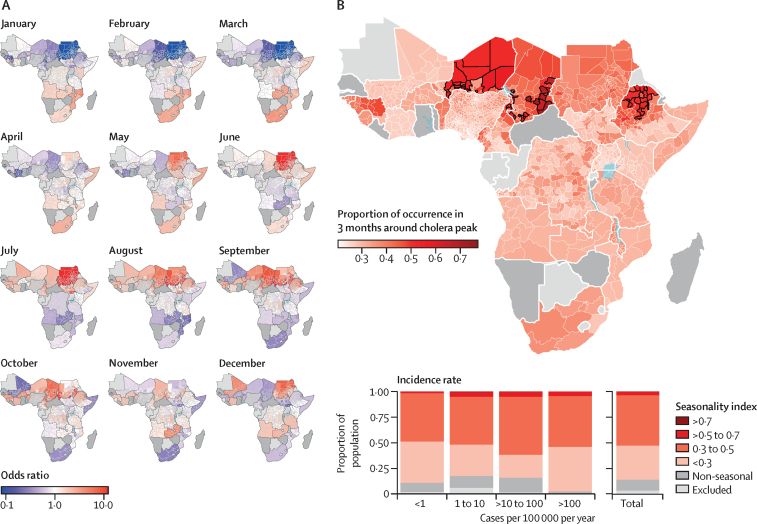
Seasonality patterns and strength of cholera occurrence in sub-Saharan Africa (A) Estimates of monthly odds ratios of excess cholera occurrence, defined as monthly incidence greater than the 2010–16 baseline;^3^ estimates are given for the best-performing model at the first or second administrative levels, depending on data availability (appendix pp 1–3), as opposed to countries for which the non-seasonal null model was selected (dark grey) and countries that were excluded from analyses due to absence of data (light grey); for illustrative purposes, the odds ratios colour scale was truncated at ±2·5 on the log scale (0·08–12·00 on the natural scale); country-level odds ratios time-series are shown in the appendix (p 11). (B) Strength of cholera seasonality quantified as the proportion of risk occurring in the 3-month window around the seasonal cholera peak. Administrative units for which the majority (at least 50%) of the probability of excess cholera occurred in the 3-month window around the peak are highlighted (black borders).

Cholera seasonality showed distinct regional patterns (figure 1, appendix pp 11–12, 14). West Africa generally had higher excess risk of cholera occurrence between July and October, particularly for inland countries across the Sahel, except for Mali. For example, the far north of Cameroon had a 10·0-times (95% credible interval [CrI] 4·3–20·0) increase in the odds of excess cholera in October compared with average odds throughout the year. In southern Africa and Mozambique, excess cholera risk generally peaked between December and April. Within East Africa, seasonal cholera patterns were more heterogeneous. Countries of the Great Lakes region and Angola had weak seasonality, peaking in the second half of the calendar year, with Kenya and Tanzania having December-to-January peaks. South Sudan and Somalia had similar seasonality patterns, with peak cholera risk occurring between May and July. Finally, Ethiopia had a distinct north–south divide, with the north peaking in August–September and the south having a weaker seasonal signal.

The strength of seasonality, quantified as the proportion of cholera risk in the 3 months surrounding the risk peak, varied within and between countries (figure 1B, with uncertainty shown in appendix p 13). Seasonality was strongest in the Sahel and northern Ethiopia. Most of the SSA population live in areas that reported cholera 30–50% of the time within the 3-month period surrounding the risk peak. The proportion of the population living in low seasonality areas (less than 30% of reporting in the 3-month window) decreased as mean annual cholera incidence rates increased from fewer than one case per 100 000 population per year to 100 cases per 100 000 per year and then decreased to similar levels as the total population for more than 100 cases per 100 000 per year. Overall, the strength of seasonality did not have a clear association with the population size, population density, area, or mean annual incidence at the administrative unit level (appendix p 13).

**Figure 2:**
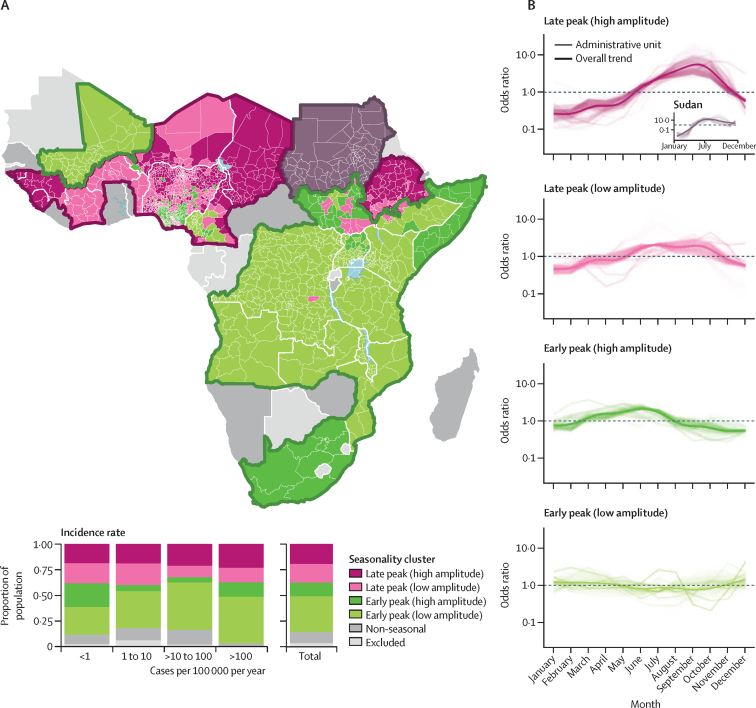
Seasonality grouping in sub-Saharan Africa (A) Map of cholera seasonality clusters (top) and proportion of population in each cluster for different categories of mean annual cholera incidence (bottom); clustering results are shown for models with three (thick borders) and five (colour fill) clusters, along with countries for which seasonality was not retained (dark grey) and excluded because of no data (light grey); macroregions were outlined with use of the convex hull of the corresponding administrative units; these macroregions were based on a three-cluster model where Sudan formed its own cluster. (B) Seasonality of cholera excess risk in each cluster for each administrative unit and overall trend estimated by a generalised additive model of odds ratio as a function of the month of the year.

Cholera seasonality patterns across SSA are clustered into two distinct macroregions that partition SSA into: western plus the Sahel; and eastern plus southern Africa (figure 2). On one hand, the macroregion consisting of western Africa and the Sahel, including northern Ethiopia and excluding Mali, had higher risk of excess cholera in the second half of the calendar year, between July and October (late peak in figure 2). On the other, cholera tended to peak at the beginning of the year in eastern and southern Africa and with less pronounced seasonality (early peak in figure 2). Across analyses with different numbers of clusters, Sudan consistently formed its own cluster due to the strong, albeit uncertain, seasonality, although its shape was similar to those surrounding areas with late and high amplitude peaks (figure 2B). Model comparison indicated statistical support for at least three groups, with similar levels of support for more than three clusters (appendix p 15). Increasing the number of clusters led to the partitioning of these two macroregions in groups of administrative units with similar shapes but different amplitude (appendix p 16). In this study, we focused on clustering results with five groups composed of two degrees of seasonality amplitude in both macroregions, in addition to the Sudan cluster (figure 2). Areas with a late seasonal peak and strongest amplitude were in the northern Cameroon–Chad region, northern Ethiopia, and Guinea. Late peak and weaker seasonality was mostly identified in central Ethiopia and west Africa. Most of the early peak macroregion were found to follow the lower amplitude pattern in central and eastern Africa, with areas with higher amplitude mostly found in South Sudan, Uganda, Somalia, and South Africa. The repartition of people at risk of cholera among the three clusters (figure 2A) echoed results based on the seasonality index (figure 1B). The largest fraction of the SSA population lives in administrative units with low amplitude seasonality (35% of the population), and the proportion of the population in clusters with stronger seasonality tended to increase with mean annual cholera incidence rate, although this stabilises when incidence reached ten cases per 100 000 per year.

**Figure 3:**
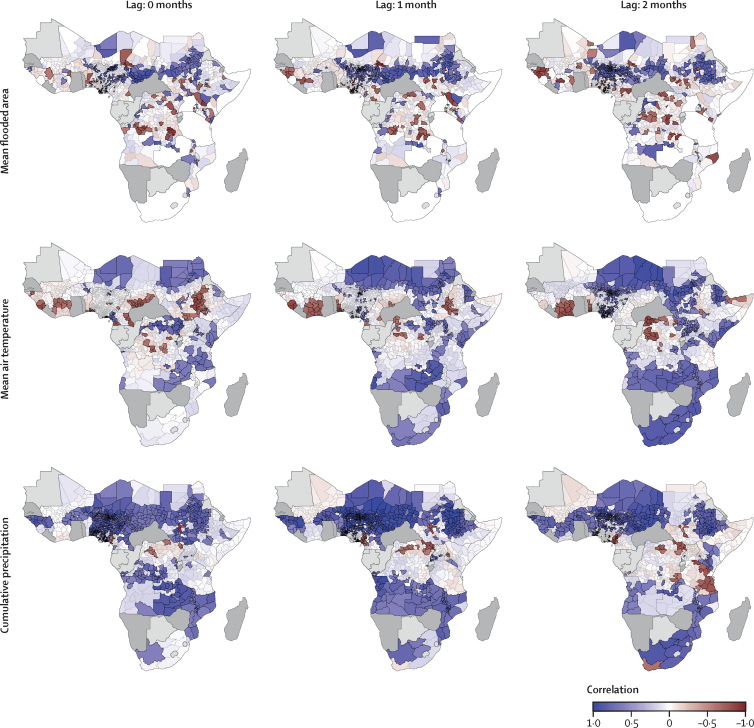
Excess cholera seasonality and climatology Maps show the Spearman correlation between odds ratios of excess cholera and the mean monthly values of hydroclimatic variables at lags of 0 months, 1 month, and 2 months. Hydroclimatic variables include the mean monthly flooded area, the mean monthly air temperature, and the monthly cumulative precipitation. Correlation is shown for significant coefficients (p value <0·05, full colour, black border), with non-significant values (p value >0·05) given for indication (transparent, no border). Associations with other hydroclimatic variables are presented in the appendix (p 18).

We explored the correlation between the monthly odds ratios of excess cholera and mean monthly fraction of area flooded, mean monthly air temperature, and cumulative monthly precipitation with lags of 0 months, 1 month, and 2 months. We found large differences in the direction and strength of these relationships across SSA (figure 3, appendix p 17). We found that the correlation between monthly excess cholera risk and flooding was generally weak in most countries and, where present, could go in both directions. We found that the spatial extent of areas where excess cholera risk and mean temperature had significant correlation was limited, with both positive (Zambia and northeastern Democratic Republic of the Congo) and negative (eastern Ethiopia, northern Côte d’Ivoire, and southern Chad) associations, although the spatial extent and strength of correlation increased with the monthly lags, particularly in southern Africa. Excess cholera risk was most consistently associated with rainfall, showing mostly strong positive correlation across SSA (71·6% of administrative units in countries with seasonality in SSA had correlations of 0·5 or higher). We identified two geographical areas with large positive correlation around lake Chad (Niger, Nigeria, northern Cameroon, and Chad), in Sudan, and in eastern Ethiopia and the central part of eastern Africa (Malawi, Mozambique, and Zambia). Associations with other hydroclimatic variables and lags are presented in the appendix (p 18).

## Discussion

In this study, we found that more than 85% of people in SSA live in locations with significant seasonal variations in excess cholera occurrence, although patterns of seasonality varied between and within countries. Seasonality was stronger in west Africa than in eastern and southern Africa, with the largest seasonality strength occurring in the Sahel. These differences mapped to macroregions with distinct seasonality patterns, within which varying degrees of seasonality amplitude were observed. Seasonality patterns correlated most consistently and strongly with mean monthly precipitation, with areas of high correlation in the countries bordering lake Chad and in southeastern Africa. Taken together, these findings suggest that cholera can be considered a seasonal disease in most sub-Saharan countries, having mixed associations with hydroclimatic factors.

Our results support the characterisation of cholera epidemiology from local analyses across countries in SSA.^9^, ^25^, ^26^, ^27^, ^28^, ^29^ The identification of areas with synchronous patterns of cholera occurrence has been highlighted before in western Africa^30^ and in the Great Lakes region.^11^ By combining data at the regional level, we showed that cholera occurrence seasonality is aligned at larger spatial scales with two main synchronous regions: one composed of west Africa and northern east Africa, and one composed of east and southern Africa. However, we note that the alignment of our estimates of seasonality does not imply synchrony of outbreaks due to the importance of their multiple drivers—including human introductions of pandemic *V cholerae,* changes in vulnerability, and changes in the immune landscape—and extrinsic factors such as natural disasters in explaining the strong inter-annual variability of cholera epidemics.^4^, ^5^ Nevertheless, the availability of large-scale estimates of seasonality provides an opportunity to formulate hypotheses on driving mechanisms as complements to local-scale studies, including pathogen introductions, variations in transmission, changes in sociobehavioural factors, and seasonal patterns of water use.

The correlations between hydroclimatic variables and the seasonality of cholera occurrence contribute to the complex picture of cholera and climate in SSA. On one hand, the absence of widespread positive correlation with mean monthly temperature, especially in all coastal areas and around large inland lakes, is further evidence against the predominant role of *V cholerae*'s environmental dynamics in driving cholera outbreaks in the region,^5^ which would imply spatial synchrony between seasonality and temperature (the Moran effect). On the other, the presence of large areas of positive correlation with cumulative monthly precipitation supports previous country-specific findings across the subcontinent.^10^, ^11^, ^25^, ^30^ These correlations hint towards the role of rainfall-driven faecal contamination of water sources in cholera occurrence, as has been suggested for different settings.^31^, ^32^, ^33^ However, our estimates are only correlations between the monthly odds ratio of excess cholera and mean monthly hydroclimatic variables, which should not be interpreted causally. The relationship between seasonal cholera occurrence and climatic drivers has also been suggested by the indirect role of the El Niño southern oscillation: the spatial distribution of cases in SSA from western to eastern Africa between El Niño and non-El Niño years, through differential sensitivity to climate anomalies.^34^ In the absence of a conceptual framework of the relationship between cholera and climate, further investigation would benefit from explicit hypotheses on the role of hydroclimatic variables on cholera occurrence, as well as accounting for possible non-linearities in their effects and interactions with social factors.^35^

Our results come with several limitations. Data used to define cholera occurrence consisted largely of suspected cholera cases due to the small proportion of suspected cases that are laboratory confirmed across SSA and the world.^14^ The definition of suspected cholera cases might vary between settings, but typically follow the WHO and GTFCC recommended case definitions.^14^ Moreover, suspected cholera reports might overestimate or underestimate true cholera infections differently in both space and time. As such, our cholera seasonality estimates might be confounded by the seasonality of other causes of watery diarrhoea. Cholera incidence estimates used to define excess occurrence (ie, months with cases higher than the mean monthly incidence) correspond to the period of 2010–16, whereas most of the cholera data used in this analysis spanned the years 2000 to 2021, which might alter the classification of cholera presence and absence. Seasonality estimates and the main results of the analysis remained qualitatively similar in sensitivity analysis using alternative definitions of cholera occurrence (one or more suspected case and ten or more suspected cases; appendix p 19). Finally, an assumption in the analysis is that seasonality did not change during the study period.

Despite these limitations, our work has public health implications from the perspective of the GTFCC's 2030 Cholera Roadmap.^36^ The presence and strength of seasonality in cholera occurrence provides an opportunity for timing preventive interventions and preparedness activities for outbreak response in periods of low odds of occurrence. The country and subnational level estimates of seasonality produced in this study can serve as a basis for these efforts, as well as for identifying outbreaks that occur before the expected season start.

## Data sharing

Code and analysis datasets consisting of monthly excess cholera, which are needed to reproduce the main findings, are available online (https://github.com/HopkinsIDD/cholera_seasonality_ssa/).

## Declaration of interests

We declare no competing interests.
